# Motor cognition in plants: from thought to real experiments

**DOI:** 10.1007/s40626-023-00304-1

**Published:** 2024-01-29

**Authors:** Bianca Bonato, Umberto Castiello, Silvia Guerra, Qiuran Wang

**Affiliations:** Department of General Psychology (DPG), University of Padova, Padua, Italy

**Keywords:** Plant cognition, Plant behavior, Kinematics, Climbing plants, Motor cognition, Cognition, Pea plants

## Abstract

Motor cognition involves the process of planning and executing goal–directed movements and recognizing, anticipating, and interpreting others’ actions. Motor cognitive functions are generally associated with the presence of a brain and are ascribed only to humans and other animal species. A growing body of evidence suggests that aneural organisms, like climbing plants, exhibit behaviors driven by the intention to achieve goals, challenging our understanding of cognition. Here, we propose an inclusive perspective under motor cognition to explain climbing plants’ behavior. We will first review our empirical research based on kinematical analysis to understand movement in pea plants. Then, we situate this empirical research within the current theoretical debate aimed at extending the principles of cognition to aneural organisms. A novel comparative perspective that considers the perception–action cycle, involving transforming perceived environmental elements into intended movement patterns, is provided.

## 1 Introduction

Motor cognition refers to processes that blend cognitive and motor functions in a seamless, interwoven fashion. Such functions evolve in space and time at various levels of complexity. The concept of motor cognition embraces the notion that cognition is embodied in action, defined as an agent’s movements to achieve a specific motor goal or in response to a meaningful event in the physical and social environment (e.g., competitive and cooperative contexts; Jeannerod 2006). Motor cognition encompasses the processes involved in planning, preparing, and producing one’s actions and the cognitive processes involved in anticipating, predicting, and interpreting others’ actions. The fundamental unit of the motor cognition paradigm is action, defined as the movements produced to satisfy an intention toward a specific motor goal. The process of motor cognition is best understood in the perception–action cycle, which involves transforming perceived environmental elements into patterns of intended movement.

An empirical example linking animals’ movement and cognition is provided by the discovery of mirror neurons in the macaque monkey’s ventral premotor and parietal cortices (Di Pellegrino et al. 1992; Rizzolatti and Craighero 2004). These neurons fire both when the animal carries out a goal–directed action and when it observes the same action performed by another individual. In humans, common neural activation during action observation and execution has also been well documented. A variety of studies have demonstrated that a motor resonance mechanism in the premotor and posterior parietal cortices occurs when participants observe or produce goal–directed actions (Amoruso and Urgesi 2016; Becchio et al. 2012; Betti et al. 2022; Edwards et al. 2003; Fadiga et al. 1995; Rizzolatti et al. 2014; Sartori et al. 2013a,b).

Naturally, ascribing motor cognition to plants is challenging because they are commonly perceived as still organisms. But plants do indeed move and interact with other individuals and their surroundings in a variety of ways (Brody and Trewavas 2023; Calvo et al. 2020; Darwin and Darwin 1880; Girloy and Trewavas 2023; Karban 2008; Kumar et al. 2020; Marder 2012; Ninkovic et al. 2021; Trewavas 2009; 2017; Trewavas et al. 2020; Wang et al. 2021a, b).

The main difficulty in perceiving and being aware of plant movements is linked to the timescales on which plants operate, which makes their movement invisible to the human eye, except in a few cases (e.g., *Mimosa pudica* L. and the *Dionea musipula* L.). Should the “perception” issue be an insurmountable problem in assigning to plant movement a component that goes beyond biomechanical constraints and relates to why an action is performed? If plant movement were examined on a time scale similar to ours, we could perceive it and understand why that specific action has been performed. By using time–lapse techniques, we might better understand plants’ behavior in terms of planning and control. After all, are we not slowing down the recording of footage of animals to achieve the same goal?

**Fig. 1:**
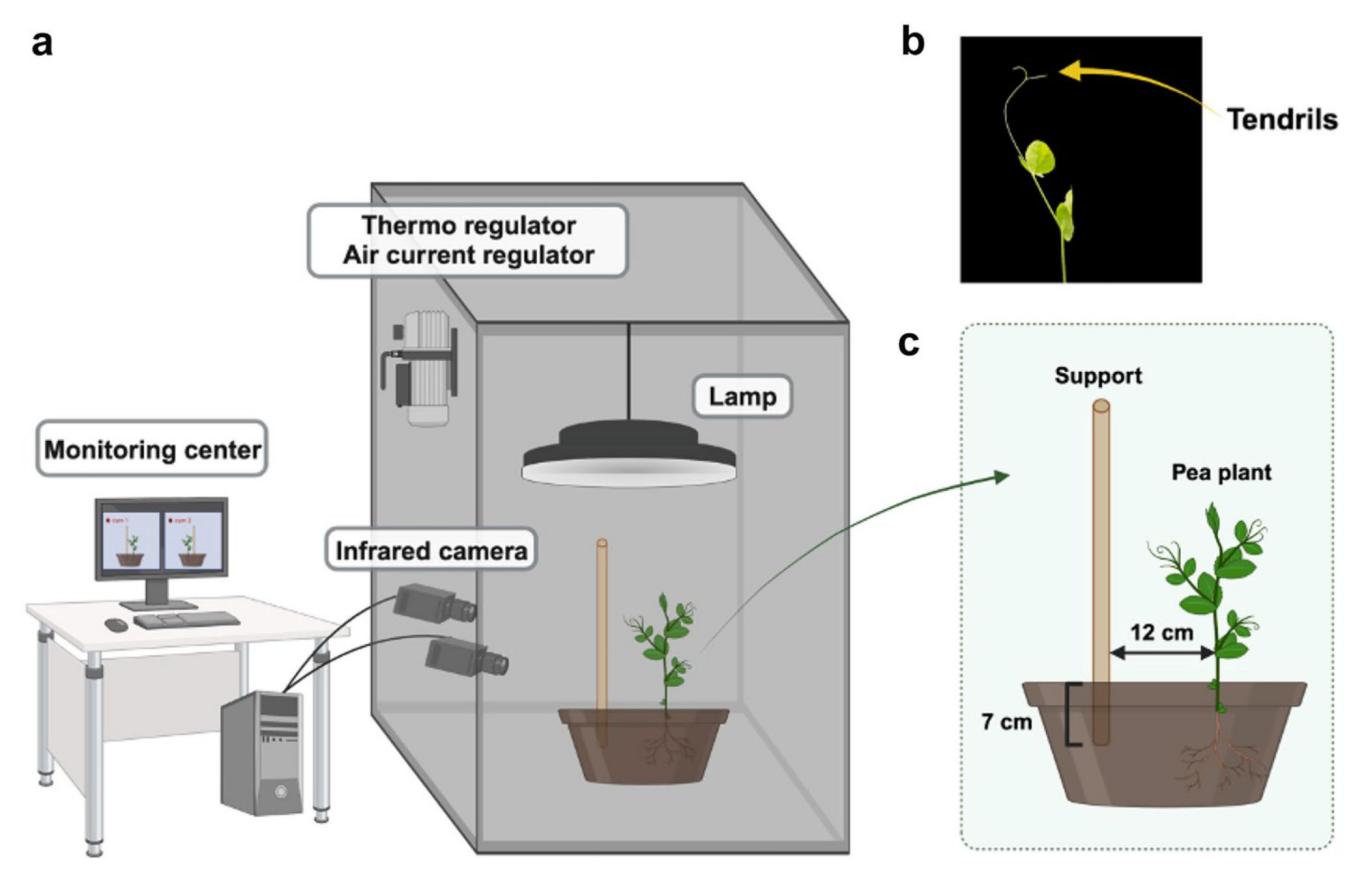
Graphical illustration of the experimental setup (**a**). Each chamber has two infrared cameras on one side, a thermoregulator for controlling the temperature, two fans for input and output ventilation, and a lamp. (**b**) The anatomical landmark of interest, namely the “tendrils,” is the primary focus of our studies. (**c**) A schematic of how *P. sativum* plants were potted together with the support in a typical situation

Here, we report on a series of three–dimensional (3D) kinematics studies, tracking and analyzing plants’ movement through time and space using dedicated in–house software (Fig. 1a–c; Simonetti et al. 2021). Evidence from these studies may shed light on the cognitive principles guiding movement planning and control in climbers, with specific reference to pea plants (*Pisum sativum* L., from here on *P. sativum*; Bonato et al. 2023, 2024; Ceccarini et al. 2020a,b; Guerra et al. 2019, 2021, 2022; Simonetti et al. 2021; Wang et al. 2023a,b). Our approach is based on the proposition that plants could be included in the “comparative” debate by capitalizing on paradigms and ideas already used to find cognitive intersections among organisms belonging to various species. The intention is not to reclassify plants as animals but to adopt effective analogies to compare cognitive abilities underlying the organization of behavior in plants and animals.

In this review, we shall begin by introducing phenomena (e.g., circumnutation; Darwin and Darwin 1880) and concepts (i.e., goal–directedness) that we will refer to throughout this review and that are necessary to understand the nature of movement in plants. After defining these key concepts, we shall illustrate our work. Through the 3D kinematical analysis of plant movement, we demonstrate that it is possible to unveil traces of cognitive processes, such as anticipatory behavior, decision–making, and social cognition. Last, we will situate our empirical findings within the perception–action cycle characterizing the concept of motor cognition.

### 1.1 Circumnutation: just a matter of rotation?

Charles Darwin and his son Francis (1880) studied the movements of several plant species. During their observations, they noticed a universal pattern of movement among plants (Darwin and Darwin 1880; Kitazawa et al. 2005), termed *circumnutation*, a movement of a plant’s growing portions to form spirals, irregular curves, or ellipses. Climbing plants, for instance, perform circumnutations to explore the environment to find potential supports (Agostinelli et al. 2021; Darwin 1875; Darwin and Darwin 1880; Gianoli 2015; Raja et al. 2020; Stolarz 2009).

Darwin (1875) stated that climbing plants might modulate circumnutation according to the structural properties of the support, as evident in *P. sativum* plants, exhibiting flexible tendrils’ responses (Bonato et al. 2023; Ceccarini et al. 2020a,b; Fukano and Yamawo 2015; Guerra et al. 2019, 2021, 2022; Sato et al. 2018; Smith et al. 2021; Wang et al. 2023a,b). Tendrils (i.e., filamentary organs sensitive to contact and used exclusively for climbing) tend to assume the shape of whatever surface they come in contact with, giving the impression of progressively “coding” potential supports’ features (Darwin 1875; Palm 1827; von Mohl 1827). In this case, the tendrils’ movement clearly shows that plants can modulate their behavior purposefully to achieve their goals. Also, this ability makes them an ideal model for studying how plants program and control their movement in response to various contexts to satisfy their needs. But can tendril movements be defined as goal–directed? Are *P. sativum* plants or climbing plants generally able to anticipate and respond to the changing states of the environment, or do they simply react passively to environmental elements? To answer these questions, it is necessary to properly define the notion of goal–directedness, which is the fundamental concept underlying our empirical work.

### 1.2 The concept of directedness

Cognition is for *doing*, not for *thinking* (Pezzulo 2008). To do is a matter of action, and action is defined as a *goal–directed* when it is driven by an expectation that it is likely to bring about a desired outcome. This point is crucial because it is tied to intentions. For example, to achieve a goal, grasping an object with various purposes, an agent must plan and execute a reaching and grasping action sequence not only considering the object’s structural features but also why the action has been performed, in simple terms with a *motor intention* (Becchio et al. 2010; Searle 1981). Thus, the concept of goal constitutes what motor intentions represent: goals and means to achieve those goals. In the time domain, the intention of doing a particular act precedes its actual motor execution (Pacherie 2008).

In the following sections, we will present our research based on 3D kinematical analysis, demonstrating an exquisite form of intentionally driven motor planning and control in *P. sativum* plants. These studies reveal that the movements these plants exhibit do not result from a primary cause and effect mechanism. Still, they are driven by an “intentional” component to achieving their goals.

### 1.3 Adapting to the thickness of stimuli

**Fig. 2:**
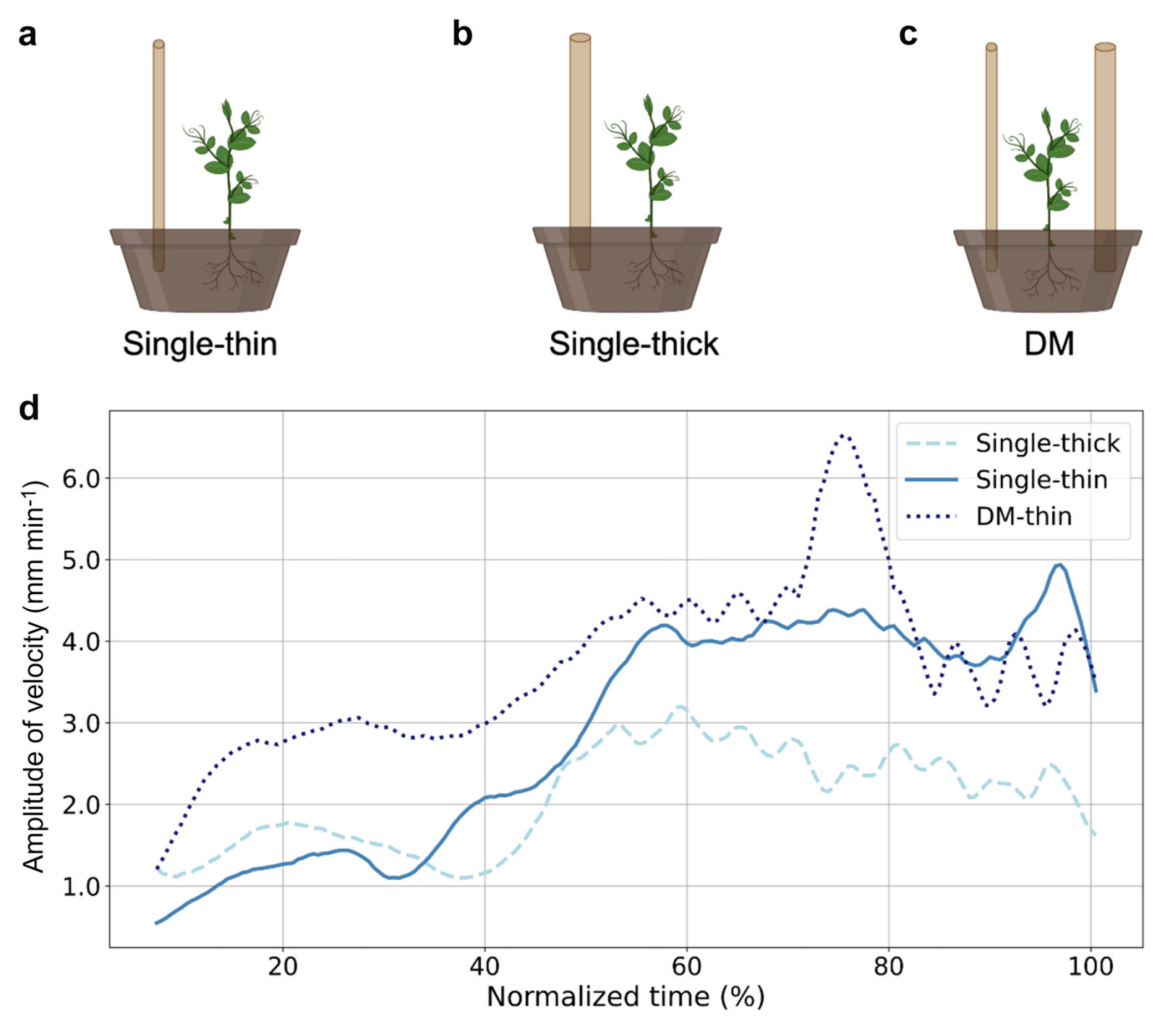
Graphical representations of the experimental setup for the **a** single–thin, **b** single–thick (Guerra et al. 2019), and **c** double–support [Decision–Making (DM); Wang et al. 2023a, b] conditions. Please note that for the DM condition, in which a thin and a thick support were presented to the plants, they preferred the thin support; therefore, the plants for the DM condition are those approaching and grasping the thin support in the presence of a larger one. **d** The velocity profile of the tendrils across conditions in absolute time (single–thin = solid line, single–thick = dashed line, DM–thin = dotted line)

Climbing plants are a suitable model for studying goal–directed behavior due to their innate capacity to detect and grasp support. One interesting question is whether they exhibit the capability to adjust their approaching and clasping movements in response to properties of the to–be–grasped support, as Charles Darwin (1880) documented anecdotally. In a recent investigation, Guerra, and colleagues (2019; see also Bell 1958; Silk and Hubbard 1991) unveiled *P. sativum* plants’ ability to detect the presence of potential support in their environment and plan a movement based on its thickness and dimensionality (Fig. 2a, b). They examined *P. sativum* plants’ approaching and grasping movements in a condition lacking potential support, a condition in which a support of a different thickness (i.e., thin or thick) or the ungraspable picture of a support was presented [i.e., two–dimensional (2D) pictures of either a thin or thick support]. The results showed that when the plants perceived the presence of the support, they rapidly changed the direction of their circumnutating movement to approach and grasp it. *P. sativum* plants adjusted the kinematics of their approaching and grasping movement in terms of their tendrils’ velocity (Fig. 2d) and aperture (i.e., the maximum distance between the tips of the tendrils) depending on support thicknesses. Plants moved more quickly and opened their tendrils more in the presence of a thinner support. When no support or the 2D picture was presented, plants circumnutated, searching for a support. When they could not find it, the tendrils stopped circumnutating and the plant collapsed due to the inclination caused by the circumnutate movement and the absence of support. The latter “photograph” condition indicates that plants can discriminate between graspable and ungraspable supports. These findings provided, for the first time, the kinematical characterization of the approaching and grasping movement in *P. sativum* plants and demonstrated their ability to assess and evaluate environmental information and transform the sensory input into complex motor behaviors (Guerra et al. 2019).

One may ask why climbing plants should vary their kinematic patterning depending on the thickness of the support. A reasonable hypothesis is that the metabolic cost of morphological modulation and circumnutations may vary. Darwin (1875) observed that climbing plants exhibit an aversive reaction toward certain supports. This effect is described with regard to *Bignonia capreolata* L. tendrils, which initially seized and then let go of sticks that were too thick. Climbing plants prefer thin supports due to various factors, such as their mechanical stability, resource allocation, and growth strategies (Darwin 1875; Darwin and Darwin 1880; Gianoli 2015; Putz 1984). In the wild, lianas are generally most abundant in early successional habitats with thin diameter supports (DeWalt et al. 2010; Ladwig and Meiners 2010; Putz 1984). This preference suggests that these plants might have evolved sophisticated mechanisms to make decisions to maximize their chances of survival (Smith et al. 2021).

In recent years, research on plant decision–making has increased, revealing that their ability to make decisions is no longer a hidden phenomenon (Dener et al. 2016; Gruntman et al. 2017; Saito 2022; Shemesh et al. 2010; Wang et al. 2023b). Severino (2021) posited that plants exhibit intelligent decision–making abilities and suggested that formulating and testing hypotheses about these decisions may be a valuable approach for investigating complex phenomena that cannot be fully explained through the prevailing mechanistic paradigm. For instance, Wang and colleagues (2023b) conducted a study to understand the principles underlying support searching in *P. sativum* plants. They examined circumnutation kinematics in plants exposed to either a single support (single condition) or two supports (Decision–Making condition—DM; Fig. 2c) of different thicknesses. The results show that plants prefer thinner supports. In addition, the kinematical patterning varied depending on whether they were exposed to one or two potential supports. When exposed to two supports, they moved more quickly and executed fewer but larger circumnutations (Fig. 2d). This could signify that while aiming at thinner supports, alternatives determine a decisional complexity played out in the kinematics of circumnutation. Finally, the results suggest that *P. sativum* plants’ movement is driven by the isochrony principle: maintaining the movement constant and scaling velocity throughout to cover longer distances. These novel observations provide further information on how plants decide to move toward a particular support and how this decision plays out in the kinematics of their movement.

### 1.4 Speed–accuracy trade–off

An action necessitates the covariation of speed and accuracy to be performed appropriately. This phenomenon is known as the speed–accuracy trade–off (Fitts 1954; Fitts and Peterson 1964). The time required to complete an action is proportional to the information needed to regulate the movement. We explored whether plants could modify the velocity and time of their reaching and grasping movements toward a support demanding varying degrees of accuracy (Ceccarini et al. 2020a). The results showed that plants can sense and process the support’s features and strategically modulate the velocity and duration of their approaching movement concerning the thickness of the stimulus. In the presence of a thick support, *P. sativum* plants decrease their average and the maximum tendril velocity during their approaching and grasping movement. Then, movement time was shorter for the thinner than for the thicker stimulus. A slower approaching movement may allow the plants to acquire more information about the thick support, considered a more demanding task, and implement corrective adjustments to reduce the possible risk of errors. The reduced velocity may permit modulation and correction of the trajectories for a more accurate selection of the contact points to twine around the support. These findings revealed that plants can plan and execute an action mediated by action–effect anticipations (Chittka et al. 2009; Franks et al. 2003; Heitz and Schall 2012).

Accuracy is another important aspect of climbers’ behavior. A movement is generally characterized by two phases: an initial movement of the effector toward the target and a deceleration stage under the supervision of the on–line control system, allowing for the added benefit of monitoring and occasionally adjusting movement in flight.

In the latter phase, the movement is refined, improving its accuracy (Ceccarini and Castiello 2018; Novak et al. 2002) through corrective adjustments (i.e., submovements), which reduce any spatial discrepancy between the effector and target position (Fradet et al. 2008). In the presence of a task requiring more precision, more secondary movements are performed to reduce the endpoint variability of an effector, hence the probability that the effector fails to grasp the target successfully (Meyer et al. 1988). We explored whether this motor principle also applies to plants and whether *P. sativum* plants have developed a motor accuracy mechanism to improve their movement’s accuracy and reduce the probability of errors (Ceccarini et al. 2020b). *P. sativum* plants’ approaching movements toward either a thin or a thick support were analyzed by considering the number of submovements performed in the support’s proximity and the variability of the tendrils’ position at the end of the movement. Our findings demonstrated that plants produced more submovements in the presence of thicker than thinner supports, confirming that climbers found thicker supports more demanding (Ceccarini et al. 2020b). Therefore, it seems that these plants can use motor–correction mechanisms to process the characteristics of the stimulus and improve the accuracy of their movement, as reported in humans and other animal species (Ceccarini et al. 2020b; Meyer et al. 1988).

### 1.5 Social cognition

**Fig. 3:**
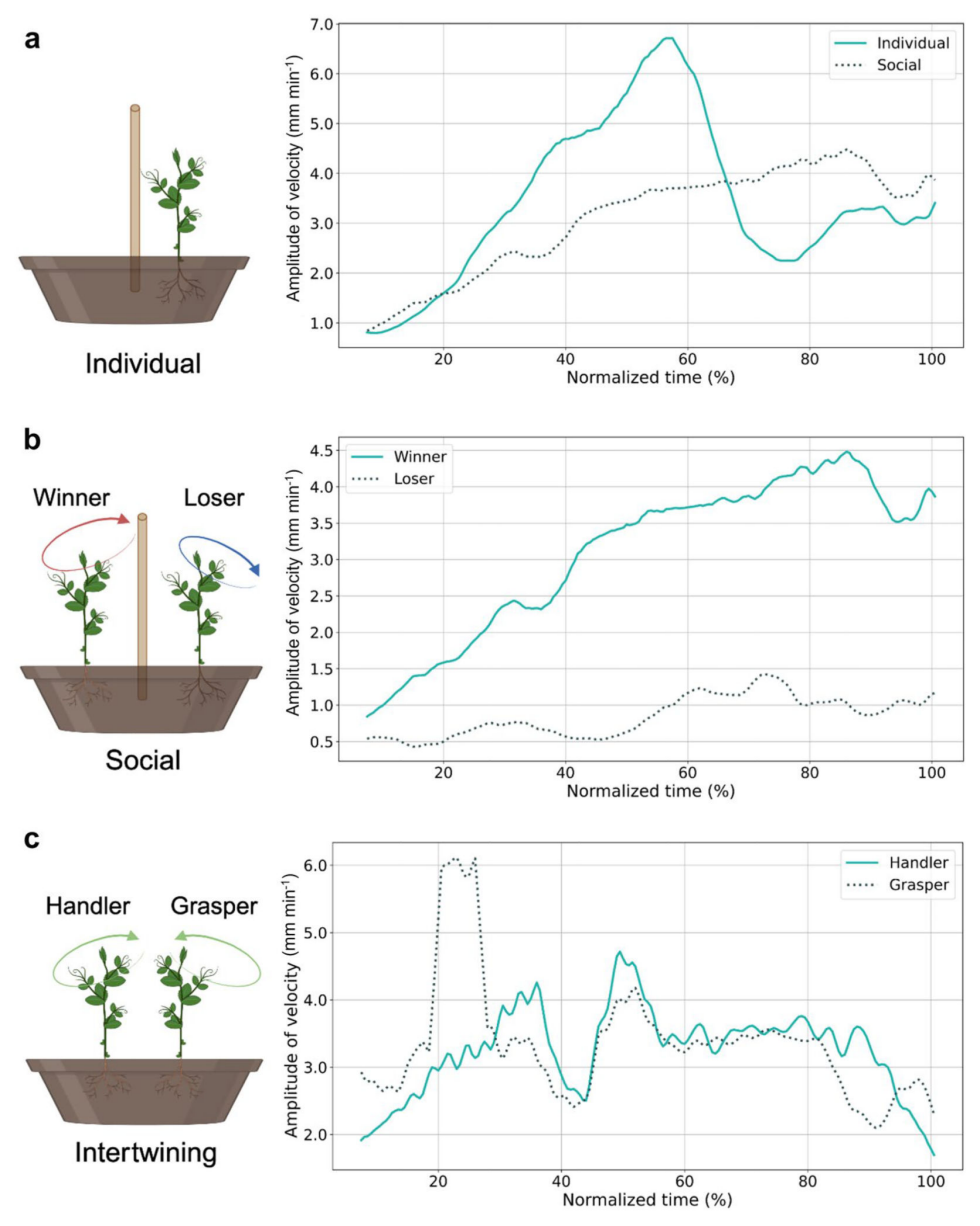
Graphical illustrations of the experimental setup and the variations in peak velocity from Bonato et al. (2023) for each comparison: **a** individual (solid line) vs social condition (dotted line), **b** winner (solid line) vs loser plant (dotted line), and from Bonato et al. (2024) **c** handler (solid line) and grasper plants (dotted line)

To act in a goal–directed manner is not only a matter of implementing the most suitable action based on the physical properties of the objects in the environment. Indeed, how an agent performs an action is a matter of biomechanical constraints and depends on the agent’s intention (i.e., “why” the action is performed). With this in mind, Bonato and colleagues (2023) investigated whether the organization of climbing plants’ kinematics is sensitive to the “intention” driving their movement toward a potential support. They investigated plants in two settings already used in humans to study individual and social motor intentions (Becchio et al. 2008, 2010; Georgiou et al. 2007): an individual or a social context (Fig. 3a, b). For the individual condition, plants acted in isolation to reach toward and grasp a potential support. For the social condition, two plants were put in the same pot with a potential support in the middle. These are both intentional actions toward the same object to grasp and the same reach–to–grasp movement to perform. The critical difference is in the “intentional” component. Whereas grasping a support requires a purely individual intention, acting in the presence of another plant inevitably involves a social intention (i.e., the intention to affect a conspecific organism’s behavior as part of one’s reason to act). The results revealed specific motor patterns for individually intended actions and actions motivated by a social intention (Fig. 3a). These results may be interpreted as evidence of the influence of intentions on kinematics, so actions embedded in different contexts show different kinematic characteristics. In comparing individual and social actions, more cautious kinematic patterning for the social situation became evident.

For instance, we are in the presence of a more careful approaching phase when the goal is situated within a social interaction. Of relevance, this occurs despite the shape, thickness, and location of the support for the individual condition matching the location, shape, and thickness of the support for the social condition. More importantly, this occurs despite no physical difference emerging in the reach–to–grasp phases across the two conditions. These findings follow what was found in humans for similar conditions (Becchio et al. 2010; Georgiou et al. 2007; Knoblich et al. 2011; Obhi and Sebanz 2011; Sebanz et al. 2003).

Looking deeper into the peculiar behavioral characteristics of the two plants acting in the social conditions, Bonato and colleagues (2023) distinguished two specific behavioral attitudes. A plant, named *winner*, exhibits a higher velocity and a time–saving approach to minimize behavioral efforts. A *loser* plant is characterized by submissive behavior with a lower velocity orienting its behavior far from the support as soon as the defeat was perceived to invest more energy in a new search (Fig. 3b). The one that grasps the support shows a perfect opposite kinematical pattern of the one that fails to attach itself to the support. This signifies that also for plants, the best strategy for time and energy saving depends on what others are doing.

Furthermore, Bonato and colleagues (2024) examined how two *P. sativum* plants coordinated their movements in time and space in the absence of potential support in the environment to achieve a common goal (i.e., support each other in the absence of potential support, therefore reaching the greatest exposure to the light; Fig. 3c). Two (or more) agents can coordinate their movement to achieve a common goal through *joint actions* (Knoblich et al. 2011; Obhi and Sebanz 2011). To act in concert during joint actions, agents must solve several coordination problems. For example, initiators of the joint action must make their intentions intelligible to their partners to establish a shared intentionality. Shared intentionality is an evolutionary response to the problems encountered during the coordination of a complex joint action that humans (Levinson 2006; Tomasello et al. 2005, 2014) and nonhuman social animals, which are capable of intricate and organized cooperation (Clutton–Brock 2009; Gelblum et al. 2015; Heesen et al. 2017, 2021; Trivers 1971), can operationalize. In this connection, we investigated whether plants could act jointly and whether some forms of shared intentionality form the basis of their “intertwining” behavior (Bonato et al. 2024; Fig. 3c). The results revealed that in the absence of a potential support, *P. sativum* plants perceived each other as external support and then acted in concert by showing specific but complementary kinematical patterns: a handler plant, the initiator of the joint action, bends exaggeratedly toward the grasper to facilitate intertwining for traveling together toward the light. The fact that one plant bends towards the other is an index of complementary behavior aimed at facilitating the intertwining process. A grasper plant exhibits a classic circumnutation pattern perpendicular to its axis and strategically modifies its tendrils’ trajectory to clasp those of the handler. Each plant seems to play a specific role, suggesting that this is not an imitative behavior, but a complementary behavior driven by a shared goal, requiring cooperation and some forms of shared intentionality (Sartori and Betti 2015; Sartori and Castiello 2013; Sartori et al. 2012; 2013a, b). In this study, the claim of shared intentionality is supported by the kinematic signatures characterizing the movement of the two plants. To meet at a precise point in space, they coordinate their action by modulating the amplitude and the timing of peak velocity. Noticeably, the movement of the two plants acting in concert differs from the one exhibited by a plant acting in isolation or towards an artificial object (i.e., a wooden pole; Bonato et al. 2024). This aspect also suggests that plants can interact differently with animate and inanimate elements in their environment.

### 1.6 How does all this happen: is it a matter of roots?

**Fig. 4:**
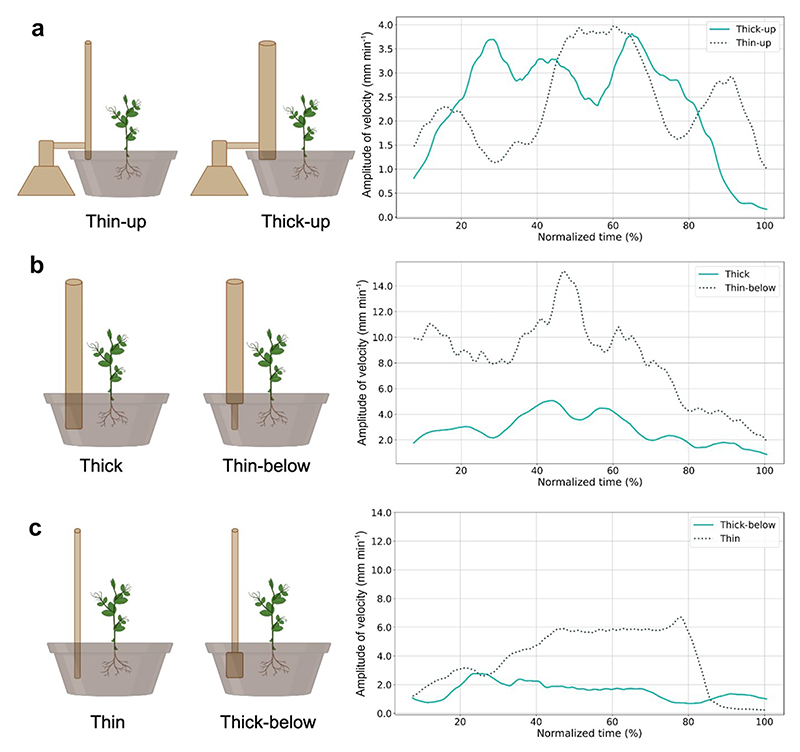
Graphical representation of the experimental conditions and the velocity profiles for each comparison. **a** In the stimulus–experiment (Guerra et al. 2021), a stimulus of different thicknesses (i.e., thin–up and thick–up) was lifted to the ground. Please note that in this scenario, plants could not perceive the presence of the potential support. The perturbation experiments (Guerra et al. 2022) with the comparisons, **b** thick vs. thin–below and **c** thin vs. thick–below, as well as the corresponding variations along the velocity profile. Note that in “b” and “c,” the growth of the upper part of the plant is driven by the size perceived below ground

At this point, the reader may wonder how *P. sativum* plants sense the presence of potential support in the environment and process its characteristics without classical sensory organs (e.g., eyes and ears) and a brain where the perceived elements are processed. A likely candidate might be the root system. Research suggests that the root system underpins several plant skills, including discovering and collecting soil nutrients and detecting surrounding plants’ presence (Cahill et al. 2010; Cahill and McNickle 2011; Dudley and File 2007; Falik et al. 2005). Mounting evidence indicates that the roots, specifically their tip (i.e., root cap), may be involved in detecting numerous signals, assessing them, and dynamically controlling the direction of root growth (Baluška et al. 2009, 2010; Falik et al. 2005; Herms et al. 2022; Trewavas 2017; Wang et al. 2021a, b). For instance, if the root tip is pressed, cut, or burnt, it transmits this information to the upper adjoining part, causing it to bend away from the affected side (Baluška et al. 2009; Darwin and Darwin 1880). Then, when the roots encounter a physical obstacle, they stop growing downwards and start growing horizontally, following the obstacle’s structure (Baluška et al. 2009; Darwin and Darwin 1880; Massa and Gilroy 2003). Thus, the roots seem able to code and process below–ground elements and behave accordingly. Guerra and colleagues (2021, 2022) exploited this phenomenon by investigating the possible contribution of the root system in supporting the thickness coding process (Fig. 4a–c). In one study, the researchers assessed the approaching and grasping behavior of *P. sativum* plants toward support of a different thickness that could be available (or not) to the root system. In particular, the support could be grounded in the soil or lifted from it (Fig. 4a). The results showed that when the support was unavailable to the root system, the plants could not locate the support and modulate the kinematical patterning of their approaching and grasping movement concerning thickness. Therefore, the results suggest that the root system is involved in sensing a support’s presence and thickness and that perceived information affects the planning and execution of the *P. sativum* plants’ approach–to–grasp movements (Fig. 4a).

However, in a natural context, what the root system finds in the soil might not be a reliable proxy for what is happening above it. For example, the roots can encounter other elements, such as rocks, which do not have an external part for the plant to climb. In this case, it could be disadvantageous for plants to rely only on the information the root system provides. Therefore, plants should have an internal mechanism to process the information from the roots, transmit it to the aerial part (or vice versa), and regulate their behavior accordingly. If the integrated information is incompatible with the goal, an adjustment should be made (remember the online control mentioned above).

A further investigation considered the functional equilibrium and interactivity between the root system and the shoot growth (Guerra et al. 2022). A group of plants was tested with support in which the belowground part was thin and the aboveground part was thick (i.e., thin–below perturbation condition; Fig. 4b), and another group was tested with the inverted condition: the support was thick below-ground and thin aboveground (i.e., thick–below perturbation condition; Fig. 4c). Control conditions, in which a single–thickness support (i.e., thin, or thick) was presented to the plant, were compared to the perturbed conditions (Fig. 4b, c). The results demonstrated that the movement duration for the perturbed trials was longer than for the control conditions. This suggests that the thickness mismatch requires more processing than a single–thickness support because more information needs to be processed. Comparing the thin–below and control–thick conditions, we found that the kinematical pattern mirrors the one observed when the unperturbed thin and the thick conditions were compared (Fig. 4b; Ceccarini et al. 2020a,b; Guerra et al. 2019, 2021). These results suggest that the movement was programmed based on the information from the support’s underground portion (i.e., the thin portion). However, comparing the thick–below and the control–thin conditions, we found no kinematical effects linked to the perturbed condition (Fig. 4c). In this case, what was programmed based on the information from the support’s underground portion (i.e., the thick portion) fits the requirements for grasping the aboveground portion (i.e., the thin portion). Indeed, for *P. sativum* plants, grasping a thicker support is a more demanding activity than grasping a thinner one (Ceccarini et al. 2020a,b; Guerra et al. 2019; Wang et al. 2023b). Therefore, it might be easier to adapt a movement pattern to grasp a thicker, more demanding support for grasping a less demanding, thinner one. The perturbation effects were thus minimized, and no differences with the control condition were found. The results indicate that the roots convey “information” to the shoot, which can regulate growth and behavior. A sort of functional equilibrium is reached through a cross–talk between the grounded and aerial components of the plant in which different signals can determine the dynamics of the tendrils for adapting to thickness (Guerra et al. 2022).

### 1.7 Motor cognition: linking data to cognitive theories

Motor cognition can be seen as the process by which an agent can gain knowledge about itself, others, or the environment through movement (Jackson and Decety 2004; Jeannerod 2006). Based on our studies, we argue that motor cognition is a theoretical paradigm that can be applied to explain the behavior of our *P. sativum* plants. Their movement seems driven by the intention to achieve a specific goal, and the intentional component impacts how the movement is planned. Kinematics is modulated by the physical properties (e.g., thickness) of a to–be–grasped support (Ceccarini et al. 2020a,b; Guerra et al. 2019, 2021, 2022; Wang et al. 2023b) and the context in which the action takes place (Bonato et al. 2023). Plants do this by implementing a series of processes remindful of motor control, decision-making, and social cognition (Bonato et al. 2023, 2024; Guerra et al. 2019, 2021, 2022; Wang et al. 2023a,b). For each scenario, the process involves sensing and interpreting environmental signals to make deliberate decisions, even when facing contradicting circumstances.

According to the classical model of cognitivism, the aspect that most characterizes cognition, and motor cognition in our particular case, is the presence of mental representations implying the presence of a central nervous system (CNS). In this view, organisms without a brain should not be able, in principle, to generate such kinds of representations. However, there is no reason to assume that cognition is intimately linked to the presence of a CNS and the ability to build mental representations (Calvo 2007; Calvo and Keijzer 2011; Bianchi and Castiello 2023; Trewavas 2003, 2005, 2009, 2014).

A criticism of the classical theory of cognition is that it focuses only on cognitive processes, neglecting the sensorimotor domain. Nowadays, however, the sensorimotor system is no longer considered a passive executive mechanism for planning and executing goal–directed behaviors. Still, it is central to a rethinking of *cognition*. Scholars who support the activist, embodied, and extended cognition theories believe that cognition is not only in the head but extends beyond the body’s limitations (Chemero 2013; Clark 2008; Gallagher 2005; Hutto and Myin 2012, 2017; Noë and Noë 2004; Thomasson 2007). They question the concept of representational content by taking extra–neural, bodily structures and the environment into account. These theories posit that a cognitive system determines the external world’s elements and/or features through its free and autonomous interaction with its surroundings rather than through representations (Varela et al. 1991). In this view, cognition is not an internal processing of information but an adaptive behavior that results from the agent’s flexible ability to deal with the environment through goal–directed actions (Bateson 1972; Maturana and Varela 1998, 1991; Parise et al. 2023; Varela et al. 1991).

In this connection, Gibson (1977, 1979) posited that cognitive operations are not exclusively dependent on mental representations but on *affordances* (i.e., opportunities for action), defined as structural supports or resources provided by the environment. According to Gibson (1977, 1979), an organism perceives an object based on its physical characteristics and affordances or what it may do with it. A single environmental element may provide an agent with multiple opportunities for action, so how does the agent choose and adopt some affordances and not others? Motivation and intention are of great importance in situations in which an environment provides multiple affordances (Gibson 1977, 1979; Withagen et al. 2012). Intentions and motivations determine which informational features are relevant and need to be attended to at any given moment, hence the salience of these affordances, which are based on the norms and rules subtending the relationship between an organism and its environment (Brancazio and Segundo–Ortin 2020a, 2020b). Indeed, the agent establishes an adaptive interaction with its environment to select and acquire the necessary environmental information to control and coordinate its behavioral responses to satisfy its needs (Gibson 1979). Moreover, the extent to which external conditions influence the resulting behavior depends on the sophistication of the organism’s ability to perceive the external signals and the environment’s affordances. Our studies (Bonato et al. 2023, 2024; Ceccarini et al. 2020a,b; Guerra et al. 2019, 2021, 2022; Wang et al. 2023a,b) have demonstrated that plants can sense and rank the environmental elements, analyze them, retain the relevant information, and use it to behave in a complex environment (Trewavas 2003, 2017). In this view, *P. sativu* plant’s ability to explore its environment, search for potential support, and select the most appropriate one to reach the greatest light exposure (i.e., the goal) exemplifies how the concept of motor cognition translated into affordances can be applied to plants, which may therefore be considered cognitive agents by all means (Calvo 2007; Calvo and Keijzer 2011). Furthermore, when inspecting our “social cognition” studies (Bonato et al. 2023, 2024), another tenet underlying the motor cognition paradigm is evident: the ability of plants to recognize, predict, and understand the behavior of other plants to act accordingly. Remember the pattern of movement exhibited by the plants when acting competitively and cooperatively.

In this view, plants set goals and control their behaviors without the need for an internal representation. A parsimonious explanation could be that the interaction with the environment pushes the homeostasis settings to a new state, and the plant will coordinate its actions to be within these settings. The outcome is, for the observer, the appearance of complex and well controlled behavior, but for the plant, there is no representation, just the maintenance of its homeostasis. This can be seen as cognitive but not representational. It cannot be excluded, however, that plants might have the ability to build some forms of representations of what surrounds them, obviously not based on neuronal circuits, but rather, on cellular schemes or metabolic networks as recently proposed (Bianchi and Castiello 2023; Debono 2013; Souza et al. 2017, 2018). But whether they would be “equivalent” to mental representations needs further testing at both behavioral and physiological levels.

To wrap up, the empirical research conducted in our laboratory presents the potential to elucidate plant movement and situate it within a non-representational motor cognition theoretical framework. From this perspective, the term *motor cognition* can be applied to diverse phenomena that result in adaptive interactions between biological organisms and their environment (Bechtel and Bich 2021). According to this, a system is defined as cognitive when it is open to exploring its environment to meet its needs and goals—instead of simply reacting to external cues—and can actively regulate its sensorimotor coupling in context–sensitive ways.

## 2 Conclusion

The empirical characterization of plant behavior presented here is contextualized in established theories for *motor cognition* across taxa. Here, we propose a comparative approach suggesting that cognition and movement are inextricably connected, keeping in mind that even organisms that do not move (e.g., porifera, lichens, certain algae, etc.) are cognitive. Suppose we decide to examine the question of plant cognition comparatively under the umbrella of action. In that case, we can take advantage of experimental models and paradigms already utilized to study cognitively driven behavior in animals. Both plants and animals are different, but these models can facilitate our comparison of how plants and animals interact with environmental cues. What may emerge from our study of plant and animal behaviors is the realization that they complement each other nicely and, if nothing else, demonstrate how similar all free–living organisms are to one another. This is not to attribute animal–like movements to plants or anthropomorphize their behavior but to demonstrate that intentional movements can also be observed in aneural organisms. This may necessitate a rephrasing, if not a complete overhaul, of current characterizations of cognition, which rely on concepts that can sometimes be arbitrary and constraining.

However, research on plants’ behavior and “cognitive” abilities is just beginning. The experimental models and paradigms presented in this paper could be used to investigate other aspects of plants that are still hidden or poorly understood. In conclusion, all claims must be substantiated through empirical evidence, including investigations at behavioral and physiological levels. This requires the use of species–specific tests in a diversified multidisciplinary framework that remains receptive to future developments and improvements.
